# The interaction effect of bystander cardiopulmonary resuscitation (CPR) and dispatcher CPR on outcomes after out-of-hospital cardiac arrest

**DOI:** 10.1038/s41598-022-27096-9

**Published:** 2022-12-27

**Authors:** Youdong Sohn, Gyu Chong Cho, Youngsuk Cho

**Affiliations:** 1 Department of Emergency Medicine, Hallym University, Kangdong Sacred Heart Hospital, 150, Sungan-ro, Gangdong-gu, Seoul, Republic of Korea; 2 Department of Biomedical Engineering, Graduate School, College of Medicine, Hanyang University, Seoul, Republic of Korea

**Keywords:** Health care, Medical research

## Abstract

This study aimed to evaluate the effects of bystander cardiopulmonary resuscitation (CPR) and dispatcher-assisted CPR (DA-CPR) on outcomes after out-of-hospital cardiac arrest (OHCA). We conducted a prospective observational study using the Korean Cardiac Arrest Research Consortium registry database and enrolled adults aged > 20 years who sustained OHCA. The study population comprised 13,864 patients from October 1, 2015, to June 30, 2021. All enrolled patients were transported to the emergency room and resuscitated by the emergency medical personnel. Patients with terminal illnesses, pregnancy, “do not resuscitate” cards, and insufficient recorded information were excluded. Good neurologic outcomes were noted in 6.5%, 9.9%, and 9.6% of patients in the “no bystander”, “standard bystander”, and “compression-only bystander” CPR groups, respectively, and differed significantly (*p* < 0.001). Survival to discharge differed significantly (*p* < 0.001) between groups at 10.8%, 13.1%, and 13.2%, respectively. In a multivariable model, the interaction between “compression-only” and DA-CPR showed a positive effect on good neurological outcomes and survival to discharge with an odds ratio of 1.93 (Confidence interval, CI 1.28–2.91, *p* = 0.002) and 1.74 (CI 1.24–2.44, *p* = 0.001), respectively. In conclusion, the interaction between compression-only CPR and DA-CPR is significantly associated with good neurological and survival outcomes after OHCA. Education for bystanders and dispatchers should adhere to the current guidelines to improve outcomes among OHCA victims.

## Introduction

Out-of-hospital cardiac arrest (OHCA) is a global public health problem with a low survival rate. Survival rates vary between communities but generally range between 5 and 10%^1–4^. From the perspective of the prehospital system to manage OHCAs, the role of the bystander and emergency medical services (EMS) is crucial. Therefore, international guidelines highly recommend cardiopulmonary resuscitation (CPR) initiated by bystanders and continued by EMS^5–7^. However, it remains debated how bystanders can be encouraged to provide immediate CPR. Although there is no doubt that early CPR performed by bystanders is important, there are factors hindering bystanders from attempting resuscitation, such as fear, cultural aspects, and a lack of confidence regarding CPR^8–10^. Dispatcher-assisted CPR (DA-CPR) was introduced in 2010 to overcome these problems^11^. In this study, we aimed to evaluate the effects of bystander CPR methods and DA-CPR, on neurological and survival outcomes after OHCA.

## Methods

### Korean EMS system

The EMS system in Korea is a public fire department-based system operated by the government. Partially dual-dispatch ambulances (BLS-fire engine, ACLS-ambulances) are dispatched for each OHCA case from regional EMS agencies. Emergency Medical Technicians in Korea provide basic to intermediate levels of service, equivalent to the EMT-basic and EMT-intermediate levels of the North American EMS. EMS dispatch and on-scene management are provided under the national EMS protocol. The National Emergency Management Agency has developed and implemented a DA-CPR protocol since August 2012. The protocol includes two key questions regarding mental status and respiration for recognising cardiac arrest. If the answers to both questions are “no response” and “abnormal respiration”, respectively, the dispatcher instructs callers to perform chest compression-only CPR if standard CPR cannot be performed independently. A declaration of death at the scene is prohibited unless the patient has obvious signs of death. This declaration is only to be carried out under direct medical control by an EMS physician on duty at a dispatch centre. Therefore, most patients with OHCA are transported to an eligible hospital for continuing CPR^12,13^.

### Study population

We conducted a prospective observational study using the Korean Cardiac Arrest Research Consortium (KoCARC) registry database, a multicentre OHCA patient registry from 65 hospitals in the Republic of Korea (ClinicalTrials.gov, NCT03222999). This database was developed in 2014 by a collaborative research network of clinicians to coordinate research on OHCA^14^. Researchers collected OHCA cases in a web-based electronic database registry using a standardised registry form based on the Utstein-style guidelines for the uniform reporting of cardiac arrest, including Utstein core elements and specific variables^15^. We enrolled adults aged > 20 years who had sustained an OHCA presumed to be of cardiac origin between October 1, 2015, and June 30, 2021. All enrolled patients were transported to the emergency room and resuscitated by the EMS. The exclusion criteria included patients with terminal illnesses; pregnant women; patients with “do not resuscitate” cards; cases of noncardiac OHCA caused by trauma, drowning, poisoning, burns, electrocution, or asphyxia; and those with insufficient information in their records.

### Human ethical approval and informed consent

The requirement for informed consent was by the institutional review boards (IRBs) of the participating institutions (Seoul National University Hospital, Konkuk University Medical Center, Kyung Hee University Hospital, Korea University Guro Hospital, Korea University Anam Hospital, SMG-SNU Boramae Medical Center, Yonsei University Severance Hospital, Yonsei University Gangnam Severance Hospital, Hallym University Kangdong Sacred Heart Hospital, Hallym University Kangnam Sacred Heart Hospital, Hanyang University Seoul Hospital, Kyungpook National University Hospital, Chosun University Hospital, Seoul National University Bundang Hospital, Myongji Hospital, Korea University Ansan Hospital, Dongguk University Ilsan Hospital, Bundang Jesaeng Hospital, Wonkwang University Sanbon Hospital, Hallym University Dongtan Sacred Heart Hospital, Chungbuk National University Hospital, Soonchunhyang University Cheonan Hospital, Jeju National University Hospital, Hanyang University Guri Hospital, Gyeongsang National University Hospital, Ajou University Hospital, Pusan National University Yangsan Hospital, Ewha Womans University Mokdong Medical Center, Inha University Hospital, and Hallym University Sacred Heart Hospital). However, Samsung Medical Center, Asan Medical Center, Wonju Severance Christian Hospital and Incheon St. Mary’s Hospital received informed consent from individual participants for the follow-up survey of neurological outcomes and death. (The requirement for informed consent was waived for participants who did not need a telephone interview for the follow-up survey.) This study was approved by the Ethics Committee of Kangdong Sacred Heart Hospital (No. 2015–09-002). All methods were carried out in accordance with KoCARC research guidelines and regulations. In addition, the researchers used anonymised data and did not have access to any personal identifiers of the individuals in the registry.

### Outcome variables

The primary outcome was the comparison among groups depending on the method of bystander CPR received: no bystander CPR, standard bystander CPR, or compression-only bystander CPR. The no bystander CPR group consisted of individuals who did not receive immediate chest compressions by bystanders at the scene. The standard bystander CPR group consisted of those who received chest compressions and ventilation from bystanders. The compression-only bystander CPR group consisted of those who received chest compressions without ventilation from bystanders.

The secondary outcomes were neurologic outcomes at discharge and survival to discharge. Neurologic outcomes were divided into good neurologic outcomes and poor neurologic outcomes, defined by CPC score. Good neurologic outcomes refer to a CPC score of 1 (good recovery) or 2 (moderate disability) on the five-category scale. Poor neurologic outcomes refer to a CPC score of 3 (severe disability), 4 (a vegetative state), or 5 (death)^16,17^.

### Statistical analysis

The participants were divided into three groups according to the bystander CPR method received: “no attempt”, “standard” CPR, and “compression-only” CPR. Groups were compared using the chi-squared test for categorical variables and analysis of variance tests for continuous variables. Continuous variables were tested for normality using the Shapiro–Wilk test. A multivariable logistic regression analysis was conducted to estimate the effect of each bystander CPR method on neurological and survival outcomes, and the results were expressed as adjusted odds ratios (ORs) with 95% confidence intervals. The regression model was adjusted for the potential confounders of age, sex, history of diabetes and hypertension, the bystander CPR method, the attachment of an AED pad, place, type of initial ECG, and the provision of dispatch CPR. Interaction analyses were performed to investigate the relationship between bystander CPR method and the provision of DA-CPR. We used the Akaike information criterion (AIC) model selection to compare the multivariable analyses with and without the interaction. R (version 4.0, R Foundation for Statistical Computing) was used for all computational analyses. Statistical significance was defined as a two-tailed P-value of < 0.05.

## Results

**Figure 1:**
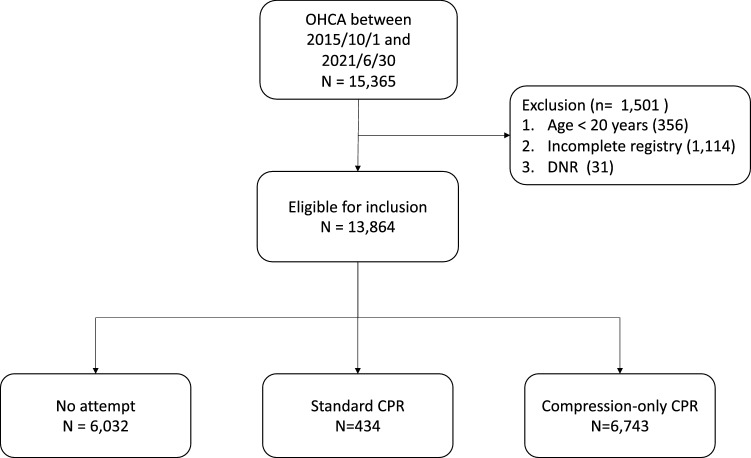
Diagram of the study OHCA, Out-of-hospital cardiac arrest; DNR, do-not-resuscitate; CPR, Cardiopulmonary resuscitation.

During the study period, 15,365 cardiac arrests occurred. Of these, 1501 cases were excluded because of blow age criteria, incomplete registry, and advanced directives. Finally, 13,864 patients met the inclusion criteria (Fig. 1). The study population was predominantly elderly, with more males than females (65.4%). The population had a low prevalence of diabetes and hypertension. “Standard” CPR, defined as performing chest compressions and delivering ventilation, was rarely started. On the contrary, half of the population received “compression-only” CPR. Most individuals resided in residence facilities. The automated external defibrillator (AED) was rarely applied at the scene. A shockable rhythm on the initial electrocardiogram (ECG) was observed in 18.5% of the study population, which consisted of 2367 cases of ventricular fibrillation and 81 cases of pulseless ventricular tachycardia compared to those of a non-shockable rhythm (3045 cases of pulseless electrical activity, 7758 cases of asystole). DA-CPR was provided for 73.6% of the study population. A good cerebral performance category (CPC) score of 1 or 2 and survival upon hospital discharge accounted for 8.1% and 12.1% of the population, respectively. This study adhered to the registered protocol. Nevertheless, some variables had missing values, ranging from 0.4 to 4.7% of the variable’s data (Table 1).

**Table 1:** General characteristics of study population.

Variables		Missing	stats
Age	Mean ± SD	0 (0.0%)	68.5 ± 15.4
Sex	Female	0 (0.0%)	4798 (34.6%)
	Male		9066 (65.4%)
Diabetes	No	0 (0.0%)	10,731 (77.4%)
	Yes		3133 (22.6%)
Hypertension	No	0 (0.0%)	9455 (68.2%)
	Yes		4409 (31.8%)
Bystander witness	No	0 (0.0%)	6057 (43.7%)
	Yes		7807 (56.3%)
Bystander CPR	No CPR	655 (4.7%)	6032 (45.7%)
	Standard CPR		434 (3.3%)
	Compression-only CPR		6743 (51.0%)
AED	No	49 (0.4%)	12,662 (91.7%)
	Yes		1153 (8.3%)
Place	Non-residence	238 (1.7%)	4929 (36.2%)
	Residence		8697 (63.8%)
Initial ECG	Non-shockable	613 (4.4%)	10,803 (81.5%)
	Shockable		2448 (18.5%)
DA-CPR	No	288 (2.1%)	3579 (26.4%)
	Yes		9997 (73.6%)
CPC*	Poor	0 (0.0%)	12,740 (91.9%)
	Good		1124 (8.1%)
Survival to discharge	No	0 (0.0%)	12,184 (87.9%)
	Yes		1680 (12.1%)

*CPR* Cardiopulmonary resuscitation; *AED* Automated electrical defibrillator; *ECG* Electrocardiography; *DA-CPR* Dispatcher-assisted CPR; *CPC* Cerebral Performance Category.

*A CPC score of 1 or 2 was considered a good neurological outcome, and a score of 3, 4, or 5 was considered a poor neurological outcome.

**Figure 2:**
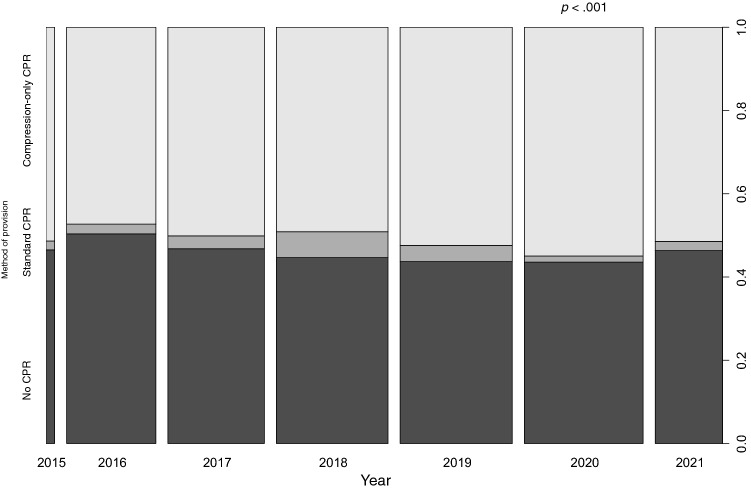
The spine plot of the association test for the bystander CPR methods performed over the years. The horizontal axis chronologically describes the study period, with the width of the bar indicating the proportion of participants each year compared to the total number of participants. The vertical axis shows the bystander CPR method. There was a statistically significant difference in the proportion of compression-only CPR over the years compared to no bystander CPR and standard bystander CPR (*p* < .001).

We investigated the association between bystander CPR performance and time (Fig. 2). The proportion of patients in the “compression-only” bystander CPR group significantly increased over the years (*p* < 0.001) compared to that in the no bystander CPR group and the standard bystander CPR group.

Table 2 shows statistically significant differences among the three groups for all variables except for sex and medical history of diabetes. The “standard” bystander CPR group and “compression-only” CPR group were more likely to receive DA-CPR and have favourable clinical outcomes compared to that of the no bystander CPR group.

**Table 2:** Comparison among groups depending on the method of CPR.

Variables		No bystander CPR (N = 6032)	Standard bystander CPR (N = 434)	Compression-only CPR (N = 6743)	*p*
Age	Mean ± SD	69.1 ± 15.1	68.1 ± 15.8	68.2 ± 15.6	.006
Sex	Female	2078 (34.4%)	155 (35.7%)	2332 (34.6%)	.865
	Male	3954 (65.6%)	279 (64.3%)	4411 (65.4%)	
Diabetes	No	4672 (77.5%)	340 (78.3%)	5206 (77.2%)	.835
	Yes	1360 (22.5%)	94 (21.7%)	1537 (22.8%)	
Hypertension	No	4186 (69.4%)	297 (68.4%)	4510 (66.9%)	.010
	Yes	1846 (30.6%)	137 (31.6%)	2233 (33.1%)	
Bystander witness	No	2693 (44.6%)	144 (33.2%)	2927 (43.4%)	< .001
	Yes	3339 (55.4%)	290 (66.8%)	3816 (56.6%)	
AED	No	5266 (87.4%)	391 (90.1%)	6450 (95.7%)	< .001
	Yes	758 (12.6%)	43 (9.9%)	289 (4.3%)	
Place	Non-residence	2332 (39.2%)	190 (44.7%)	2188 (32.8%)	< .001
	Residence	3624 (60.8%)	235 (55.3%)	4482 (67.2%)	
Initial ECG	Non-shockable	4954 (86.1%)	288 (75.4%)	5141 (78.2%)	< .001
	Shockable	801 (13.9%)	94 (24.6%)	1436 (21.8%)	
DA-CPR	No	2733 (46.7%)	61 (14.4%)	594 (8.9%)	< .001
	Yes	3113 (53.3%)	362 (85.6%)	6084 (91.1%)	
CPC*	Poor	5641 (93.5%)	391 (90.1%)	6098 (90.4%)	< .001
	Good	391 (6.5%)	43 (9.9%)	645 (9.6%)	
Survival to discharge	No	5380 (89.2%)	377 (86.9%)	5852 (86.8%)	< .001
	Yes	652 (10.8%)	57 (13.1%)	891 (13.2%)	

*CPR* Cardiopulmonary resuscitation; *AED* Automated electrical defibrillator; *ECG* Electrocardiography; *DA-CPR* Dispatcher-assisted CPR; *CPC* Cerebral performance category.

*A CPC score of 1 or 2 was considered a good neurological outcome, and a score of 3, 4, or 5 was considered a poor neurological outcome.

We performed a logistic regression analysis to adjust for potential confounding factors from the prehospital Utstein elements on good neurologic outcomes and survival upon discharge (Tables 3 and 4). The multivariable model revealed that the “compression-only” CPR group had less favourable outcomes than those of the no bystander CPR group and the provision of DA-CPR had less favourable outcomes as well. However, in an interaction model, there was a significantly positive interaction when “compression-only” CPR by bystanders and DA-CPR were performed simultaneously. The interaction models with good neurological and survival outcomes showed lower AIC scores than the models without an interaction. Therefore, we selected an interaction model to explain this phenomenon.

**Table 3:** Logistic regression on the effect of good neurologic outcomes showing odds ratio.

Variables		Poor CPC* (N = 11,459)	Good CPC (N = 958)	Model 1 (univariable)	Model 2 (multivariable)	Model 3 (multivariable with interaction)
Age	Mean ± SD	69.8 ± 15.0	56.0 ± 13.5	0.95 (0.94–0.95, *p* < .001)	0.96 (0.96–0.97, *p* < .001)	0.96 (0.96–0.97, *p* < .001)
Sex	Female	4109 (35.9%)	183 (19.1%)			
	Male	7350 (64.1%)	775 (80.9%)	2.37 (2.01–2.79, *p* < .001)	1.07 (0.88–1.30, *p* = .487)	1.07 (0.88–1.31, *p* = .472)
Diabetes	No	8743 (76.3%)	841 (87.8%)			
	Yes	2716 (23.7%)	117 (12.2%)	0.45 (0.37–0.55, *p* < .001)	0.67 (0.53–0.85, *p* < .001)	0.67 (0.53–0.85, *p* = .001)
Hypertension	No	7738 (67.5%)	694 (72.4%)			
	Yes	3721 (32.5%)	264 (27.6%)	0.79 (0.68–0.92, *p* = .002)	1.48 (1.23–1.79, *p* < .001)	1.48 (1.22–1.79, *p* < .001)
Bystander witness	No	5341 (46.6%)	171 (17.8%)			
	Yes	6118 (53.4%)	787 (82.2%)	4.02 (3.39–4.76, *p* < .001)	2.20 (1.81–2.67, *p* < .001)	2.22 (1.83–2.70, *p* < .001)
Bystander CPR	No CPR	5224 (45.6%)	345 (36%)			
	Standard CPR	337 (2.9%)	31 (3.2%)	0.93 (0.64–1.36, *p* = .716)	0.71 (0.45–1.10, *p* = .125)	1.07 (0.44–2.61, *p* = .888)
	Compression-only CPR	5898 (51.5%)	582 (60.8%)	0.67 (0.58–0.77, *p* < .001)	0.82 (0.68–0.99, *p* = .036)	0.77 (0.55–1.09, *p* = .141)
AED	No	10,577 (92.3%)	839 (87.6%)			
	Yes	882 (7.7%)	119 (12.4%)	1.70 (1.39–2.09, *p* < .001)	1.47 (1.13–1.91, *p* = .004)	1.43 (1.10–1.86, *p* = .007)
Place	Public	3793 (33.1%)	576 (60.1%)			
	Residence	7666 (66.9%)	382 (39.9%)	0.33 (0.29–0.38, *p* < .001)	0.65 (0.55–0.76, *p* < .001)	0.64 (0.54–0.76, *p* < .001)
Initial ECG	Non-shockable	9976 (87.1%)	180 (18.8%)			
	Shockable	1483 (12.9%)	778 (81.2%)	29.08 (24.50–34.50, *p* < .001)	17.73 (14.76–21.29, *p* < .001)	17.85 (14.85–21.44, *p* < .001)
DA-CPR	No	2912 (25.4%)	300 (31.3%)			
	Yes	8547 (74.6%)	658 (68.7%)	0.75 (0.65–0.86, *p* < .001)	0.69 (0.57–0.84, *p* < .001)	0.53 (0.41–0.70, *p* < .001)
Standard CPR x DA-CPR						0.85 (0.30–2.41, *p* = .756)
Compression-only CPR x DA-CPR						1.93 (1.28–2.91, *p* = .002)
AIC					4332	4326

*OR* Odds ratio; *CPC* Cerebral performance category; *CPR* Cardiopulmonary resuscitation; *AED* Automated electrical defibrillator; *ECG* Electrocardiography; *DA-CPR* Dispatcher-assisted CPR; *AIC* Akaike Information Criterion.

*A CPC score of 1 or 2 was considered a good neurological outcome, and a score of 3, 4, or 5 was considered a poor neurological outcome.

**Table 4:** Logistic regression on the effect of survival to discharge showing odds ratio.

Variables		No survival discharge (N = 10,987)	Survival to discharge (N = 1430)	Model 1 (univariable)	Model 2 (multivariable)	Model 3 (multivariable with interaction)
Age	Mean ± SD	70.0 ± 15.0	58.7 ± 14.5	0.96 (0.95–0.96, *p* < .001)	0.97 (0.96–0.97, *p* < .001)	0.97 (0.96–0.97, *p* < .001)
Sex	Female	3969 (36.1%)	323 (22.6%)			
	Male	7018 (63.9%)	1107 (77.4%)	1.94 (1.70–2.21, *p* < .001)	1.05 (0.90–1.22, *p* = .530)	1.05 (0.91–1.22, *p* = .500)
Diabetes	No	8375 (76.2%)	1209 (84.5%)			
	Yes	2612 (23.8%)	221 (15.5%)	0.59 (0.50–0.68, *p* < .001)	0.85 (0.71–1.02, *p* = .080)	0.85 (0.71–1.02, *p* = .082)
Hypertension	No	7402 (67.4%)	1030 (72%)			
	Yes	3585 (32.6%)	400 (28%)	0.80 (0.71–0.91, *p* < .001)	1.27 (1.09–1.48, *p* = .002)	1.27 (1.09–1.47, *p* = .002)
Bystander witness	No	5221 (47.5%)	291 (20.3%)			
	Yes	5766 (52.5%)	1139 (79.7%)	3.54 (3.10–4.05, *p* < .001)	2.27 (1.96–2.64, *p* < .001)	2.29 (1.97–2.66, *p* < .001)
Bystander CPR	No CPR	4989 (45.4%)	580 (40.6%)			
	Standard CPR	324 (2.9%)	44 (3.1%)	1.17 (0.84–1.62, *p* = .351)	0.78 (0.53–1.14, *p* = .195)	0.75 (0.33–1.67, *p* = .476)
	Compression-only CPR	5674 (51.6%)	806 (56.4%)	1.22 (1.09–1.37, *p* < .001)	1.05 (0.90–1.22, *p* = .521)	0.70 (0.52–0.94, *p* = .018)
AED	No	10,172 (92.6%)	1244 (87%)			
	Yes	815 (7.4%)	186 (13%)	1.87 (1.57–2.21, *p* < .001)	1.45 (1.18–1.79, *p* < .001)	1.43 (1.16–1.76, *p* < .001)
Place	Public	3560 (32.4%)	809 (56.6%)			
	Residence	7427 (67.6%)	621 (43.4%)	0.37 (0.33–0.41, *p* < .001)	0.66 (0.58–0.76, *p* < .001)	0.66 (0.57–0.75, *p* < .001)
Initial ECG	Non-shockable	9659 (87.9%)	497 (34.8%)			
	Shockable	1328 (12.1%)	933 (65.2%)	13.65 (12.07–15.44, *p* < .001)	8.80 (7.69–10.07, *p* < .001)	8.85 (7.73–10.12, *p* < .001)
DA-CPR	No	2756 (25.1%)	456 (31.9%)			
	Yes	8231 (74.9%)	974 (68.1%)	0.72 (0.63–0.81, *p* < .001)	0.75 (0.64–0.88, *p* < .001)	0.62 (0.51–0.76, *p* < .001)
Standard CPR x DA-CPR						1.15 (0.46–2.87, *p* = .769)
Compression-only CPR x DA-CPR						1.74 (1.24–2.44, *p* = .001)
AIC					6559	6552

*OR* Odds ratio; *CPR* Cardiopulmonary resuscitation; *AED* Automated electrical defibrillator; *ECG* Electrocardiography; *DA-CPR* Dispatcher-assisted CPR; *AIC* Akaike Information Criterion.

## Discussion

Among the three groups (no bystander CPR, “standard” bystander CPR, and “compression-only” bystander CPR) in our study, the group without bystander CPR comprised patients who were older, had fewer witnessed OHCA events, fewer shockable rhythms, less DA-CPR provision, poorer CPC, and less survival to discharge compared to those of the other groups. However, more patients received AED use once DA-CPR was initiated despite the low rate of its initiation. One hypothesis for this conflicting finding may be that those who are even reluctant to compress the chest of OHCA victims might apply AED when DA-CPR is successfully initiated.

Chest compression with ventilation has been the main component of resuscitation for OHCA victims since the early 1960s^18^. However, bystanders’ reluctance to perform CPR has increased because of the resistance associated with the need to perform mouth-to-mouth ventilation^8,19,20^. Therefore, the European Resuscitation Council and American Heart Association guidelines have introduced two possible methods for bystanders^21,22^. The first method is “standard” CPR with a 30:2 ratio of compression and ventilation. The second is “compression-only” CPR without pauses for ventilation, intended to encourage bystanders to undertake resuscitation efforts more frequently. Despite this effort, there is wide variation in outcomes across different studies. Some studies have reported that “compression-only” CPR is associated with better outcomes^17,23–25^. On the contrary, other studies have reported that “standard” CPR had better outcomes for selected patients with OHCA, such as those with a noncardiac OHCA, children, and those in whom there was a delay in CPR initiation^26,27^. Another study reported no differences in outcomes between “compression-only” CPR and “standard” CPR^28–30^. Similarly, our study showed heterogeneous outcomes. We suspect that this finding might be associated with the poor quality of CPR because one literature review claimed that the depth of compression in “compression-only” CPR might be shallower than that in “standard” CPR^10^. According to our results in Table 2, approximately half of the OHCA cases underwent compression-only CPR, and the rate of attempts at standard CPR is extremely low in Korea. The proportion of bystander compression-only CPR has been significantly increasing over time. Unfortunately, we could not obtain detailed information about the bystanders, such as whether they were trained for CPR. We assume that more attempts at compression-only CPR have been encouraged because “compression-only” CPR is simple and easy to learn for bystanders.

There is a need to increase the rate of bystander CPR, one of the critical components in the chain of survival, to provide early CPR before the EMS arrive^31^. DA-CPR programs are intended to encourage bystanders who are untrained or reluctant to perform CPR^5,32,33^. However, DA-CPR is less likely to result in favourable real-world outcomes. Our multivariate analysis also showed that DA-CPR had a statistically significant negative effect on good neurological outcomes and survival to discharge. This finding is similar to that of previous studies showing less survival or neurological benefit^12,34^. These researchers hypothesised that the primary cause of the reduced benefit was the low confidence of dispatchers for DA-CPR. Dispatchers may assist in improving the provision of bystander CPR. However, more time elapsed before CPR initiation under DA-CPR than for self-led bystander CPR because the bystander would instantly initiate CPR soon after they witnessed and identified an OHCA. Moreover, a longer detection time interval (DTI) from the call for an ambulance to the detection of OHCA by the dispatcher showed significantly poorer neurological recovery. This 30-s delay in DTI was associated with a 3% reduction in a good CPC score^35^.

The interaction between “compression-only” CPR and DA-CPR provision in our study showed a positive effect on good neurological and survival outcomes after adjusting for potential confounders, despite the absence of a positive effect of compression-only CPR or DA-CPR when considered independently. This finding is different from that of previous studies. There were three randomised controlled trials (RCTs) showing no significant difference between compression-only CPR with DA-CPR and standard CPR with DA-CPR^28,29,36^. One retrospective observational CPR study reported that standard CPR with DA-CPR was associated with good neurological outcomes^37^. These heterogeneous findings are attributable to multiple factors. First, a 30:2 compression-to-ventilation ratio has been recommended since 2005^38,39^. The above RCTs comprised a 15:2 ratio, as per previous CPR guidelines. Therefore, variability in CPR ratio followed may have a heterogeneous effect on the outcomes. Second, our study population was different from that of the above observational study, which excluded those who received “no attempt” CPR and “unwitnessed” cardiac arrests.

The rate of bystander CPR varies from 26 to 86% internationally^40^. Our study revealed that the rate of bystander CPR gradually increased to 50%, showing a statistically significant trend over the years. In addition, bystander CPR before the arrival of EMS is associated with an increased chance of survival after OHCA compared with no bystander CPR^41^. In addition, DA-CPR significantly increased the actual provision of bystander CPR^42^. For DA-CPR, compression-only CPR is strongly recommended for adults with suspected OHCA, though this recommendation is based on limited evidence^43^.

Hypertension is a well-known risk factor for cardiac diseases such as myocardial infarction and coronary artery disease. It is associated with a 2–3-fold increase in the risk of cardiac arrest^44^. Interestingly, our study revealed that hypertension was associated with good neurological and survival outcomes after adjusting for potential confounding factors, even though there was a significant negative effect on outcomes in each univariable analysis. This finding is unexpected. However, previous studies have also claimed that hypertension had a good survival outcome in univariable analysis^44–46^. In addition, another recent study has shown similar outcomes in univariable and multivariable analyses^47^. While this study could not fully explain why hypertension had a favourable effect on outcomes after OHCA, the authors explained that the result might come from the pathophysiology of hypertension itself or lifestyle changes and medication after the patient’s diagnosis of hypertension. Once they know of their hypertension, patients have a greater understanding of EMS activation, resulting in early recognition and early activation in the case of an OHCA^48^. Antihypertensive medications, such as angiotensin-converting enzyme inhibitors, beta-blockers, and calcium-channel blockers, had favourable outcomes in patients with cardiac arrest^49,50^. However, a recent meta-analysis showed that antihypertensive medications did not reduce the incidence of sudden death, although they did reduce the incidence of fatal and non-fatal myocardial infarction^51^. Therefore, a more speculative explanation is required.

## Limitations

This study has some limitations. First, there may be selection bias. Even though the KoCARC registry was managed by on-site researchers and a quality management committee, it was challenging to complete all sets of variables for each patient. All variables had 0.4–4.7% of their data missing. The top three variables with missing data were the bystander CPR method (4.7%), initial ECG at the scene (4.4%), and application of dispatcher assistance (2.1%). Second, there may be a lack of generalisability. The KoCARC registry is based on one Asian population, and the EMS system is different from that of other countries. Therefore, further evaluation should be conducted internationally. Third, there may be unmeasured bias. This registry was filled with prehospital Utstein elements, which were provided by EMS agencies; therefore, we were not able to collect all potentially relevant variables because EMS agencies were not willing to provide them. Fourth, there were significant shifts in CPR guidelines regarding the compression-to-ventilation ratio from 15:2 to 30:2 in 2005, the introduction of compression-only CPR as an option for bystanders who were unable or unwilling to perform ventilation in 2005, and the recommendation for DA-CPR in 2010. As our study population was enrolled in 2015, this major shift had no effect. However, these changes may influence the ability to compare our results with those of previous studies. Finally, this study was conducted prior to the coronavirus disease 2019 (COVID-19) pandemic but was continued through this period. During the pandemic, standard CPR was prohibited to prevent the spread of COVID-19. Therefore, this may negatively affect the preference for ventilation.

## Conclusion

We conclude that the interaction between compression-only CPR and DA-CPR is significantly associated with good neurological and survival outcomes after OHCA. However, each CPR method has an independent negative effect on outcomes. Education for bystanders and dispatchers should adhere to the current guidelines to improve outcomes among OHCA victims.

## Data Availability

The data that support the findings of this study are available from KoCARC but restrictions apply to their availability. These data were used under license for the current study and so are not publicly available. The data are however available from the corresponding author upon reasonable request and with the permission of the KoCARC.
